# The association between the C-reactive protein-to-albumin-to-lymphocyte index and retinopathy: insights from a population-based study

**DOI:** 10.3389/fnut.2025.1552020

**Published:** 2025-03-13

**Authors:** Pingping Li, Fangyu Chen, Lu Li, Jianhua Wu

**Affiliations:** ^1^Department of Eye Center, Renmin Hospital of Wuhan University, Wuhan, China; ^2^Department of Aier Eye Hospital of Wuhan University, Wuhan, China

**Keywords:** retinopathy, CALLY, inflammation, chronic disease, NHANES

## Abstract

**Introduction:**

Retinopathy is a multifactorial disease influenced by metabolism, immunity, inflammation, and other factors. The C-reactive protein-albumin-lymphocyte (CALLY) index is a novel immunonutritional score that has shown promise in various health contexts. This study aims to investigate the association between the CALLY index and retinopathy risk, and to compare its predictive performance with other established inflammatory markers.

**Methods:**

Data from 5,439 participants in the 2005–2008 National Health and Nutrition Examination Survey (NHANES) were utilized. Multivariable-weighted logistic regression was employed to assess the association between the CALLY index and retinopathy risk. Additionally, the predictive performance of the CALLY index was compared with other inflammatory markers. Mediation analysis was conducted to explore potential mediating factors in the association between the CALLY index and retinopathy.

**Results:**

Multivariable-weighted logistic regression revealed a significant inverse association between the CALLY index and retinopathy risk (OR = 0.96, 95% CI = 0.94–0.98, *P* = 0.002). Participants in the highest CALLY index quartile exhibited a markedly lower risk of retinopathy (*P* < 0.001). The CALLY index demonstrated superior predictive performance compared to other inflammatory markers, with an area under the curve (AUC) of 0.672 (95% CI = 0.643–0.701). Mediation analysis indicated that high-density lipoprotein (HDL) levels partially mediated the association between the CALLY index and retinopathy.

**Discussion:**

These findings highlight the CALLY index as a reliable, independent biomarker for retinopathy risk assessment, outperforming traditional inflammatory markers and oering potential clinical value for early identification of retinopathy in individuals with chronic diseases.

## 1 Introduction

Retinopathy is a common ophthalmic condition characterized by complex pathophysiological mechanisms involving metabolic dysregulation, chronic inflammation, and vascular dysfunction. It is strongly associated with systemic diseases such as diabetes and hypertension. Diabetic retinopathy (DR), the most common cause of retinal disease, is a leading cause of vision loss among individuals with diabetes, with its pathogenesis closely linked to inflammatory responses, oxidative stress, and microvascular complications (1–3). Currently, the global prevalence of hypertension and diabetes is continuously rising, and it is expected that the global burden of retinopathy will significantly increase in the coming decades.

Currently, biomarkers commonly used for retinopathy risk prediction include traditional markers such as HbA1c and blood pressure, as well as emerging biomarkers such as neutrophil-lymphocyte ratio (NLR), monocyte-lymphocyte ratio (MLR), and C-reactive protein (CRP) and so on (4, 5). However, these markers are typically singular and reflect only one aspect of the patient's condition. CRP, albumin (ALB), and lymphocyte count are critical biomarkers reflecting inflammation, nutritional status, and immune function, respectively. The C-reactive protein-to-albumin-to-lymphocyte (CALLY) index, a composite metric integrating these indicators, provides a comprehensive assessment of systemic inflammation and immunonutritional status (3, 6). Although the CALLY index has demonstrated prognostic value in various chronic diseases and malignancies, its application and relevance in retinopathy remain insufficiently explored. We conducted a study utilizing data from the 2005–2008 National Health and Nutrition Examination Survey (NHANES) to investigate the relationship between the CALLY index and retinopathy among U.S. adults. Additionally, high-density lipoprotein (HDL) has been shown to play a significant role in reducing inflammation, oxidative stress, and improving vascular health, all of which are key factors in the progression of retinopathy (7). We examined HDL whether levels mediate the association between the CALLY index and retinopathy. Elucidating this relationship could advance our understanding of the roles of inflammation and immunonutritional status in retinopathy pathogenesis and provide novel insights for early screening and intervention strategies.

## 2 Materials and methods

### 2.1 Data and study participants

We conducted a cross-sectional analysis using data from the 2005–2008 NHANES, a comprehensive program managed by the National Center for Health Statistics. NHANES serves as a critical resource for evaluating the health and nutritional status of the U.S. population through extensive interviews, which capture demographic, socioeconomic, and dietary information, as well as detailed medical examinations performed by qualified healthcare professionals, including assessments of blood biomarkers. This study was approved by the Institutional Review Board of the National Center for Health Statistics (Protocol #2005-06, Continuation #2005-06), and all participants provided written informed consent.

**Figure 1:**
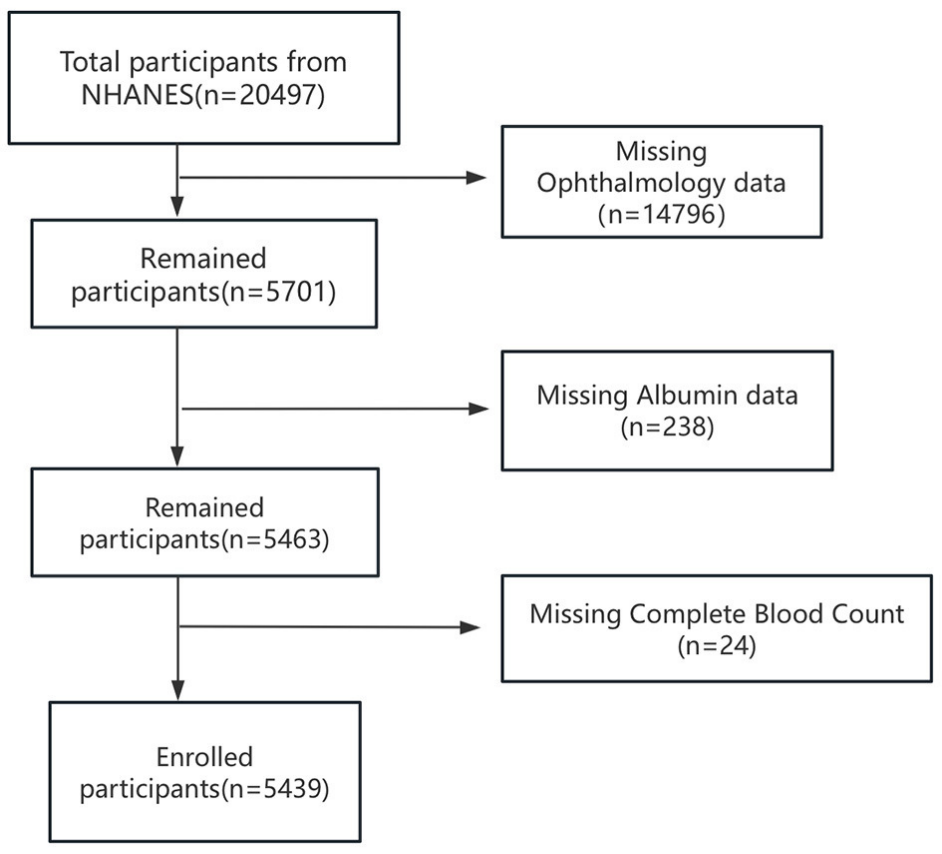
The flow diagram of the study participants, from NHANE 2005–2008.

A total of 20,497 participants were initially included. Of these, 14,796 individuals were excluded due to missing ophthalmology data, 238 due to missing serum albumin data, and 24 due to missing complete blood count data. After these exclusions, the final analytical sample comprised 5,439 participants. The detailed participant selection process is illustrated in Figure 1.

### 2.2 Date collection

Complete blood counts were analyzed using a Coulter HMX Hematology Analyzer, with results for neutrophils, platelets, monocytes, and lymphocytes reported in standardized units of × 103 cells/μL. The primary focus of our study was the CALLY index, a novel metric derived from albumin levels, lymphocyte counts, and C-reactive protein (CRP) values. This index integrates key biomarkers to provide a comprehensive assessment of systemic immunonutritional status, offering potential insights into its association with retinopathy risk. Specifically, the CALLY index was calculated as [(Albumin in g/L) ^*^ (Lymphocyte count in 1,000 cells/uL)]/(CRP in mg/dL).

Additionally, we also examined the associations of the systemic immune-inflammation index (SII), systemic inflammation response index (SIRI), monocyte-to-lymphocyte ratio (MLR), and neutrophil-to-lymphocyte ratio (NLR) with retinopathy.


$$\begin{array}{l} CALLY\;index=\frac{Albumin\;*\;Lymphocyte}{CRP}\\ \;SII\;index=\frac{Platelet\;counts\;*\;Neutrophil\;counts}{Lymphocyte\;counts}\\ \;SIRI\;index=\frac{Monocyte\;counts\;*\;Neutrophil\;counts}{Lymphocyte\;counts}\\ \;ML\;Rindex=\frac{Monocyte\;counts}{Lymphocyte\;counts}\\ \;NL\;Rindex=\frac{Neutrophil\;counts}{Lymphocyte\;counts} \end{array}$$

### 2.3 Assessment of retinopathy

Two retinal color photographs were taken for each participant using the Canon CR6-45NM Ophthalmic Digital Imaging System and the Canon CR4-45NM fundus camera: one centered on the optic disc and the other on the macular region. These images were sent to the University of Wisconsin Department of Ophthalmology for further quality assessment and grading. Retinal status was evaluated using the Early Treatment Diabetic Retinopathy Study (ETDRS) grading scale. Eyes with higher severity levels of retinopathy (grades 14–80) were classified as having retinopathy. For more detailed criteria and classifications of retinopathy diagnosis in the NHANES database, please refer to https://wwwn.cdc.gov/Nchs/Nhanes/2005-2006/OPXRET_D.htm#OPDUARMA.

### 2.4 Covariates assessment

We included demographic details, lifestyle factors, and potential confounders as covariates in the analysis. Demographic variables included age (≤ 60, >60), sex, race/ethnicity (Mexican American, non-Hispanic Black, non-Hispanic White, and other race—including multiracial), education level (less than high school, high school graduate, and college or above), and poverty-to-income ratio (PIR; ≤ 1, 1–3, ≥3). Lifestyle factors such as smoking status (SMQ040 code), alcohol consumption (ALQ101 code), and physical activity were also considered. Physical activity is defined as “moderate-intensity activity,” such as running or playing basketball, that significantly increases breathing or heart rate for at least 10 min continuously (Please refer to 2005–2006 cycle, PAD200 code, and 2007–2008 cycle, PAQ650 code). Additionally, comorbidities such as hypertension (BPQ020 code) and diabetes (DIQ010 code) were included in the analysis. The estimated glomerular filtration rate (eGFR) is calculated based on serum creatinine, with the serum creatinine (Scr) code URXCUR. The formula is as follows (8):


$$\begin{array}{l} For\;females:eGFR=144\;\times \;{\left(Scr/0.7\right)}^{\wedge }(-0.329)\;\times \;{\left(0.993\right)}^{\wedge }Age\\ \;For\;males:eGFR=144\;\times \;{\left(Scr/0.9\right)}^{\wedge }(-0.411)\;\times \;{\left(0.993\right)}^{\wedge }Age \end{array}$$

Participants with missing data >10% were excluded. Multiple imputation was used to estimate the missing data for covariates.

### 2.5 Statistical analysis

Considering the complex, stratified, and probability-based sampling design of the NHANES database, we conducted weighted analyses using WTINT2YR. Continuous variables were described as means ± standard deviations (SD), while categorical variables were expressed as percentages. The relationship between inflammatory markers and retinopathy was analyzed using univariate and multivariate logistic regression. For preliminary analyses, the continuous CALLY index was categorized into quartiles. The association between the CALLY index and retinopathy was assessed using three weighted logistic regression models: Model 1 (unadjusted), Model 2 (adjusted for sex, age, and race/ethnicity), and Model 3 (further adjusted for sex, age, race/ethnicity, education level, PIR, BMI, smoking, alcohol, hypertension, diabetes, eGFR and physical activity). The decision tree model was used to validate the quartile cutoff values of the CALLY index. The dose-response relationship between the CALLY index and retinopathy was further evaluated using restricted cubic spline (RCS) models. Subgroup analyses were conducted based on age, sex, BMI, PIR, alcohol, smoke, hypertension, diabetes, and physical activity with interaction effects assessed by the *p*-value between the CALLY index and stratification factors. Mediation analysis was performed to determine whether HDL levels mediated the association between the CALLY index and retinopathy. The total effect (TE) represented the direct association between the CALLY index and retinopathy. The indirect effect (IE) reflected the influence of HDL as a mediator, while the direct effect (DE) captured the association between the CALLY index and retinopathy after adjusting for HDL levels.

Diagnostic performance was evaluated using receiver operating characteristic (ROC) curves and the area under the curve (AUC) for the CALLY index and other inflammatory markers, including SII, SIRI, MLR, and NLR. All statistical analyses were performed using R software (version 4.2.0) and relevant R packages. A *p*-value < 0.05 was considered statistically significant.

## 3 Results

### 3.1 Baseline characteristics

In this study, participants were categorized into a retinopathy group and a non-retinopathy group based on the presence of retinopathy. Among the 5,439 participants, 50.2% were male and 49.8% were female. Compared to the non-retinopathy group, the retinopathy group had a higher proportion of males, older individuals, non-Hispanic White, participants with a poverty-to-income ratio (PIR) of 1–3, and those with a history of hypertension or diabetes. Additionally, the retinopathy group included more drinkers and individuals engaging in less physical activity. Baseline characteristics also revealed that participants in the retinopathy group had lower BMI and HDL levels, but higher CRP levels compared to those in the non-retinopathy group. In addition, NLR was significantly higher in the retinopathy group compared to the non-retinopathy group (*P* < 0.05), while no significant differences were observed for other inflammatory markers between the two groups. These differences in baseline characteristics were statistically significant (*P* < 0.05). For detailed baseline characteristics, refer to Table 1.

**Table 1:** Characteristics of the study population from NHANES 2005–2008.

	**Overall (*n* = 5,439)**	**No-retinopathy (*n* = 4,763)**	**Retinopathy (*n* = 673)**	***P*-value**
**Age (years)**	60.88 ± 12.58	60.12 ± 12.97	62.33 ± 11.71	**<0.001**
**Gender (%)**				**<0.001**
Male	50.2	49.4	56.3	
Female	49.8	50.6	43.7	
**Race/ethnicity (%)**				0.556
Mexican American	15.6	15.3	18.3	
White	54.7	56.4	43.1	
Black	19.6	18.2	28.4	
Other	10.1	10.1	10.2	
**Education level (%)**				0.349
Less than high school	14.1	13.3	19.9	
High school	14.9	14.4	18.7	
More than high school	71.0	72.3	61.4	
**Marital status (%)**				0.183
Married/living with partner	64.2	64.3	63.9	
Divorced/separated/widowed	30.0	29.0	29.9	
Never married	6.8	6.7	6.2	
**Poverty (%)**				**<0.001**
< 1	16.6	19.5	22.0	
1–3	40.9	37.8	46.5	
≥3	42.5	42.8	31.5	
**Smoking status (%)**				0.374
Every day	17.7	17.9	17.1	
Some day	2.4	2.4	2.4	
Not at all	79.9	79.7	80.5	
**BMI (kg/m** ^**2**^ **)**	28.48 ± 5.72	27.94 ± 5.88	29.56 ± 6.01	**0.050**
**Alcohol (%)**				**<0.001**
Yes	68.3	69.1	62.8	
No	31.7	30.9	37.2	
**Hypertension (%)**				**<0.001**
Yes	45.6	43.6	59.9	
No	54.4	56.4	40.5	
**Physical activity**				**<0.001**
Yes	20.2	21.0	14.3	
No	79.8	79.0	85.8	
**Diabetes**				**<0.001**
Yes	17.4	13.3	45.8	
No	82.6	86.7	54.2	
**eGFR (mL/min/1.73 m** ^**2**^ **)**	107.72 ± 15.19	108.13 ± 14.42	106.99 ± 15.70	0.06
**CRP (mg/dL)**	0.50 ± 0.89	0.47 ± 0.87	0.70 ± 1.00	**0.002**
**WBC (1,000 cells/dL)**	7.19 ± 2.30	7.19 ± 2.30	7.19 ± 2.26	0.145
**PLT (1,000 cells/dL)**	266.64 ± 69.76	267.96 ± 69.17	257.29 ± 73.21	0.094
**Monocyte (1,000 cells/dL)**	0.56 ± 0.19	0.56 ± 0.19	0.55 ± 0.18	0.112
**Lymphocyte (1,000 cells/dL)**	2.13 ± 1.22	2.13 ± 1.24	2.13 ± 1.08	0.194
**Neutrophils (1,000 cells/dL)**	4.28 ± 1.62	4.24 ± 1.64	4.53 ± 1.50	0.115
**Creatinine (mg/dL)**	41.70 ± 3.12	41.9 ± 3.06	40.26 ± 3.16	0.092
**HDL (mg/dl)**	1.38 ± 0.41	1.39 ± 0.42	1.33 ± 0.38	**0.010**
**CALLY**	749.56 ± 1251.98	801.95 ± 1299.67	378.33 ± 737.56	**<0.001**
**SII**	598.84 ± 359.01	595.93 ± 360.45	619.45 ± 348.69	0.112
**SIRI**	1.26 ± 0.87	1.25 ± 0.89	1.31 ± 0.73	0.094
**MLR**	0.29 ± 0.13	0.29 ± 0.13	0.29 ± 0.13	1.000
**NLR**	2.24 ± 1.18	2.21 ± 1.17	2.43 ± 1.20	**<0.001**

Mean ± SD for continuous variables: *P*-value was calculated by weighted linear regression.

% for categorical variables: *P*-value was calculated by weighted chi-square test. BMI, body mass index; eGFR, estimated Glomerular Filtration Rate; CRP, C-reactive protein; WBC, white blood cell; PLT, platelet; HDL, high-density lipoprotein; SII, systemic immune-inflammation index; SIRI, systemic inflammation response index; MLR, monocyte-to-lymphocyte ratio; NLR, neutrophil-to-lymphocyte ratio.

Bold indicates that the *P*-value is statistically different.

We further analyzed the relationship between the CALLY index and other inflammatory markers (SII, SIRI, MLR, NLR) with retinopathy using logistic regression. Univariate logistic regression revealed a significant association between NLR and CALLY with retinopathy (NLR: OR, 1.69; 95% CI: 1.38–1.88, *P* < 0.001. CALLY: OR, 0.95; 95% CI: 0.91–0.99, *P* < 0.001). After adjusting for other potential confounders, these associations remained significant (see detailed results in Supplementary Table S1).

### 3.2 The relationships of CALLY index and retinopathy

Among the 5,439 participants included in the study, the prevalence of retinopathy was 12.4% (673/5,439). Three models were constructed to examine the association between the CALLY index and retinopathy risk (Table 2). In Model 1, the odds ratio (OR) and 95% confidence interval (CI) were 0.95 (0.91–0.99; *P* < 0.001), indicating that each unit increase in the CALLY index was associated with a reduced risk of retinopathy. This association persisted in Model 2 (OR: 0.95; 95% CI: 0.92–0.98; *P* = 0.002) and Model 3 (OR: 0.96; 95% CI: 0.94–0.98; *P* = 0.002), even after adjusting for potential confounders. The decision tree model demonstrated that the accuracy of CALLY in predicting retinopathy was 87.9%. Please refer to Supplementary Figure S1 for details.

**Table 2:** Association of CALLY index and retinopathy.

	**Model 1**		**Model 2**		**Model 3**	
	**OR (95%CI)**	***P*-value**	**OR (95%CI)**	***P*-value**	**OR (95%CI)**	***P*-value**
Q1	Ref	—	Ref	—	Ref	—
Q2	0.56 (0.46, 0.69)	<0.001	0.57 (0.46, 0.70)	<0.001	0.63 (0.50, 0.79)	<0.001
Q3	0.41 (0.33, 0.51)	<0.001	0.43 (0.33, 0.52)	<0.001	0.44 (0.34, 0.56)	<0.001
Q4	0.18 (0.13, 0.24)	<0.001	0.19 (0.14, 0.25)	<0.001	0.24 (0.17, 0.32)	<0.001
CALLY	0.95 (0.91, 0.99)	<0.001	0.95 (0.92, 0.98)	0.002	0.96 (0.94, 0.98)	0.002
≤ 354	Ref	—	Ref	—	Ref	—
>354	0.38 (0.32, 0.45)	<0.001	0.39 (0.33, 0.46)	<0.001	0.43 (0.35, 0.52)	0.003

Model 1 was adjusted for none.

Model 2 was adjusted for age, gender and race.

Model 3 was adjusted for sex, age, race/ethnicity, education level, PIR, BMI, smoking, alcohol, hypertension, diabetes, eGFR and physical activity. OR, odds ratio; CI, confidence interval.

**Figure 2:**
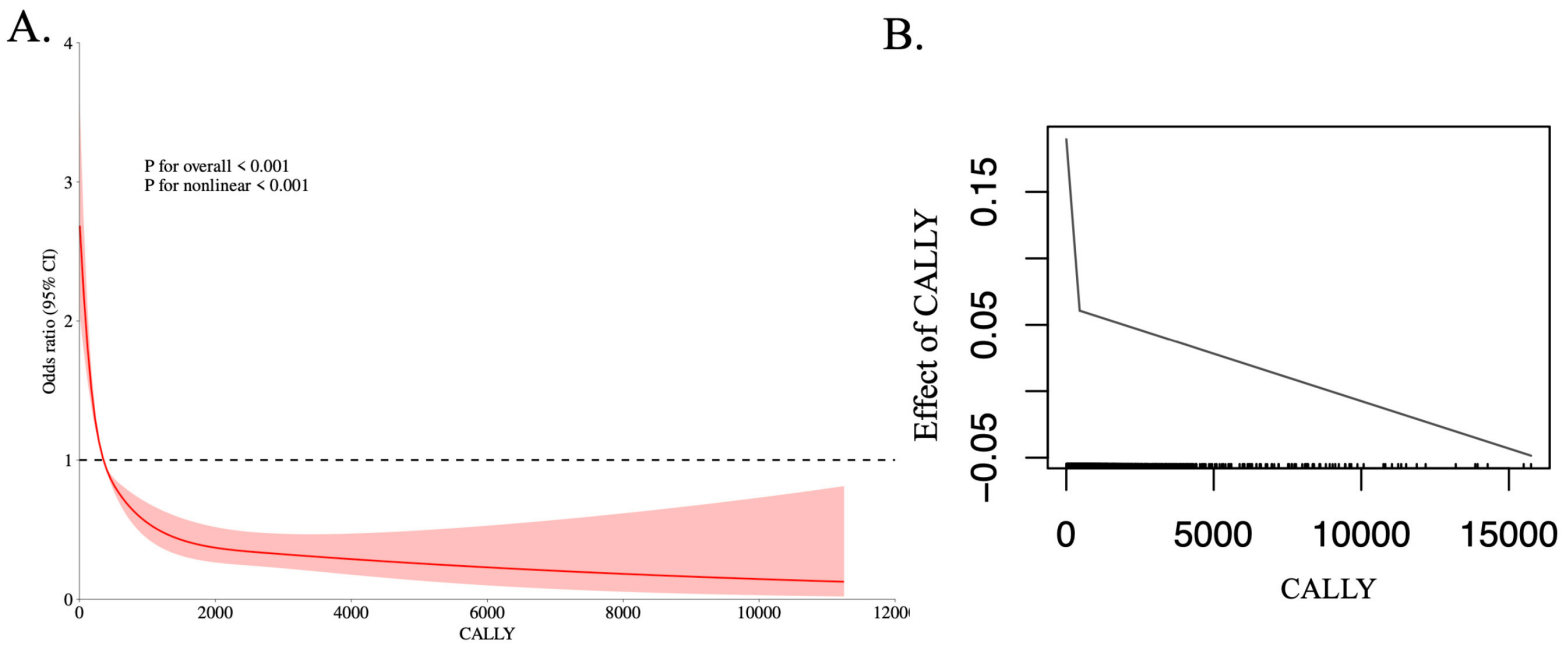
The dose-response relationship **(A)** and the two-piecewise linear regression **(B)** between CALLY and the prevalence of retinopathy.

RCS analysis revealed a non-linear relationship between the CALLY index and retinopathy risk (*P* < 0.001; Figure 2A). Further analysis using a two-piece linear regression model identified a threshold effect at a CALLY index of 354 (Table 2, Figure 2B). Specifically, participants with a CALLY index >354 exhibited a 57% lower likelihood of developing retinopathy compared to those with a CALLY index ≤ 354.

### 3.3 Subgroup analysis and interaction

The subgroup analysis revealed that the association between CALLY and retinopathy varied across different groups. A stronger association was observed in males compared to females. In terms of age, the association was significant in individuals aged over 60 years. Higher BMI and the PIR > 3 were also associated with a stronger relationship between CALLY and retinopathy. Additionally, alcohol consumption and smoking were significantly associated with the CALLY-retinopathy relationship. The association was stronger in individuals with hypertension and diabetes. Overall, sex, age, BMI, PIR, lifestyle factors (alcohol consumption, smoking), and chronic conditions (hypertension, diabetes) play important roles in modulating the relationship between CALLY and retinopathy. Detailed subgroup-specific findings are provided in Supplementary Figure S2.

### 3.4 Relationships between CALLY, and HDL

We constructed three models to perform linear analyses. The results showed that in the univariate linear regression model, CALLY was significantly positively associated with HDL levels (β = 448, 95% CI: 368–527, *P* < 0.001). Similarly, in the multivariate linear regression model adjusted for sex, age, race/ethnicity, education level, PIR, BMI, smoking, alcohol consumption, hypertension, diabetes, eGFR and physical activity, CALLY remained significantly positively associated with HDL levels (β = 530, 95% CI: 444–617, *P* < 0.001).

HDL concentrations were further categorized into quartiles. In the univariate model, participants in the Q4 group had a 34% lower risk of developing retinopathy compared to those in the Q1 group (OR 0.66, 95% CI: 0.52–0.83, *P* < 0.001). In the multivariate model, after adjusting for potential confounders, participants in the Q4 group had a 23% lower risk of retinopathy compared to those in the Q1 group (OR 0.77, 95% CI: 0.61–0.85, *P* = 0.02). For more detailed results, please refer to Tables 3, 4.

**Table 3:** Association between CALLY and HDL.

	**Model 1**		**Model 2**		**Model 3**	
	**β (95% CI)**	***P*-value**	**β (95% CI)**	***P*-value**	**β (95% CI)**	***P*-value**
CALLY	448, (368, 527)	**< 0.001**	546, (462, 630)	**< 0.001**	530, (444, 617)	**< 0.001**

Model 1 was adjusted for none.

Model 2 was adjusted for age, gender and race.

Model 3 was adjusted for sex, age, race/ethnicity, education level, PIR, BMI, smoking, alcohol, hypertension, diabetes, eGFR and physical activity. OR, odds ratio; CI, confidence interval; HDL, high-density lipoprotein.

Bold indicates that the *P*-value is statistically different.

**Table 4:** Association between HDL and retinopathy.

	**Model 1**		**Model 2**		**Model 3**	
	**OR (95%CI)**	***P*-value**	**OR (95%CI)**	***P*-value**	**OR (95%CI)**	***P*-value**
HDL (mg/dl)		**0.003**		**0.004**		**0.040**
Q1 (0.28–1.09)	Ref	-	Ref	-	Ref	-
Q2 (1.09–1.29)	0.92, (0.74, 1.14)	0.430	0.89, (0.71, 1.11)	0.290	1.05, (0.82, 1.35)	0.680
Q3 (1.29–1.60)	0.93, (0.75, 1.16)	0.540	0.91, (0.72, 1.14)	0.400	1.24, (0.96, 1.61)	0.090
Q4 (1.60–3.93)	0.66, (0.52, 0.83)	**< 0.001**	0.64, (0.50, 0.82)	**0.005**	0.77, (0.61, 0.85)	**0.030**

Model 1 was adjusted for none.

Model 2 was adjusted for age, gender and race.

Model 3 was adjusted for sex, age, race/ethnicity, education level, PIR, BMI, smoking, alcohol, hypertension, diabetes, eGFR and physical activity. OR, odds ratio; CI, confidence interval. HDL, high-density lipoprotein.

Bold indicates that the *P*-value is statistically different.

### 3.5 Mediation analysis

**Figure 3:**
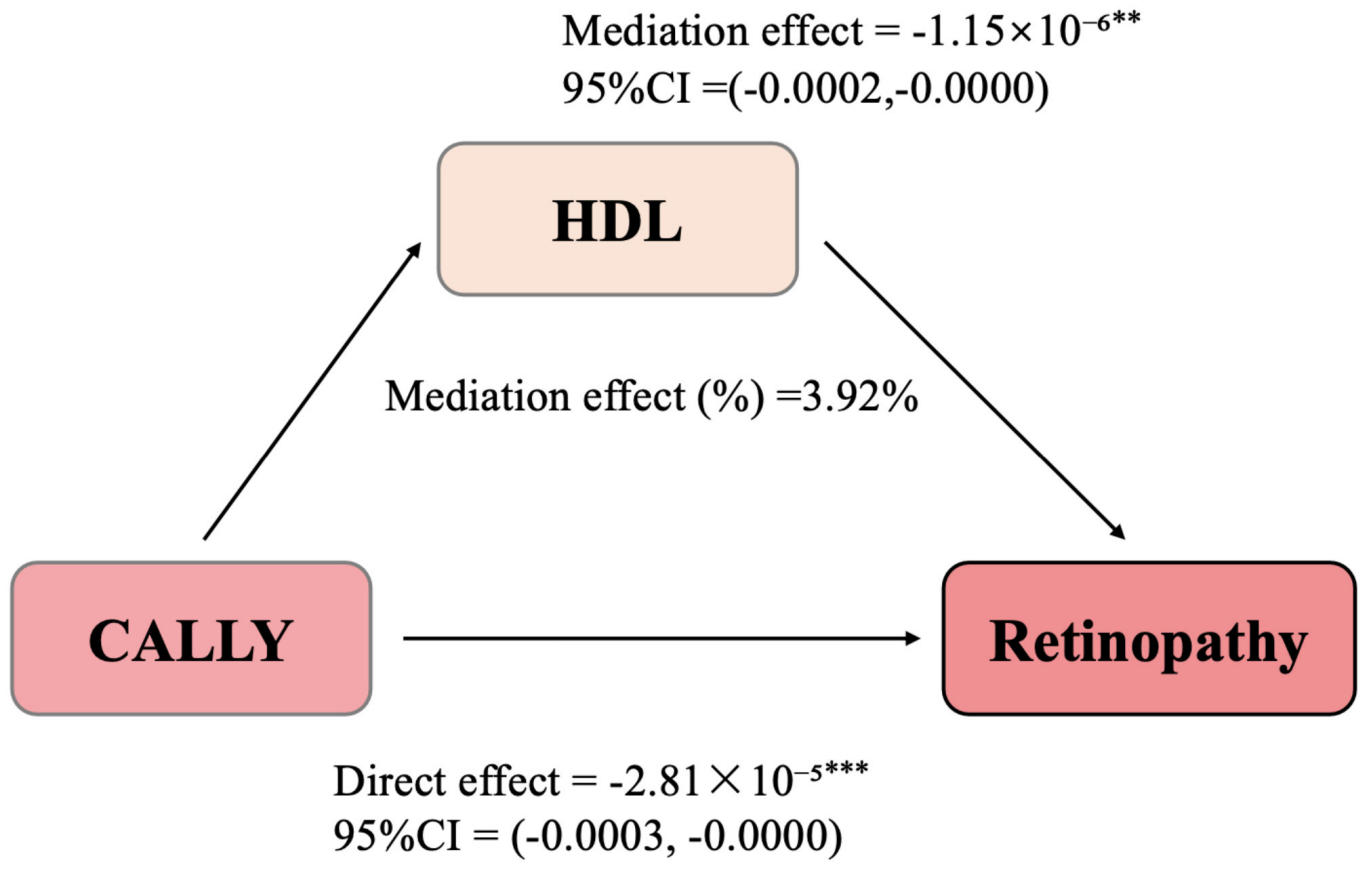
Path diagram of the mediation analysis models. HDL partially mediates the relationship between CALLY and retinopathy. ^**^Represents the *P*-value < 0.01, ^***^Represents the *P*-value < 0.001.

To further investigate whether HDL levels mediate the relationship between CALLY and retinopathy, we conducted a mediation analysis. In this model, CALLY was treated as the independent variable, retinopathy as the dependent variable, and HDL as the mediator. The confounding factors included sex, age, race/ethnicity, education level, PIR, BMI, smoking, alcohol consumption, hypertension, diabetes, eGFR, and physical activity. As illustrated in Figure 3, CALLY exerted a significant indirect effect on the occurrence of retinopathy through HDL levels, with an IE of −1.15 × 10^−6^ (95% CI: −0.0002 to −0.0000). This indicates that HDL partially mediates the relationship between CALLY and retinopathy.

Even after adjusting for HDL levels, CALLY maintained a significant inhibitory effect on retinopathy, with a DE of −2.81 × 10^−5^ (95% CI: −0.0003 to −0.0000). These findings suggest that ~3.92% of the total effect of CALLY on retinopathy is mediated through HDL.

### 3.6 ROC analysis

**Figure 4:**
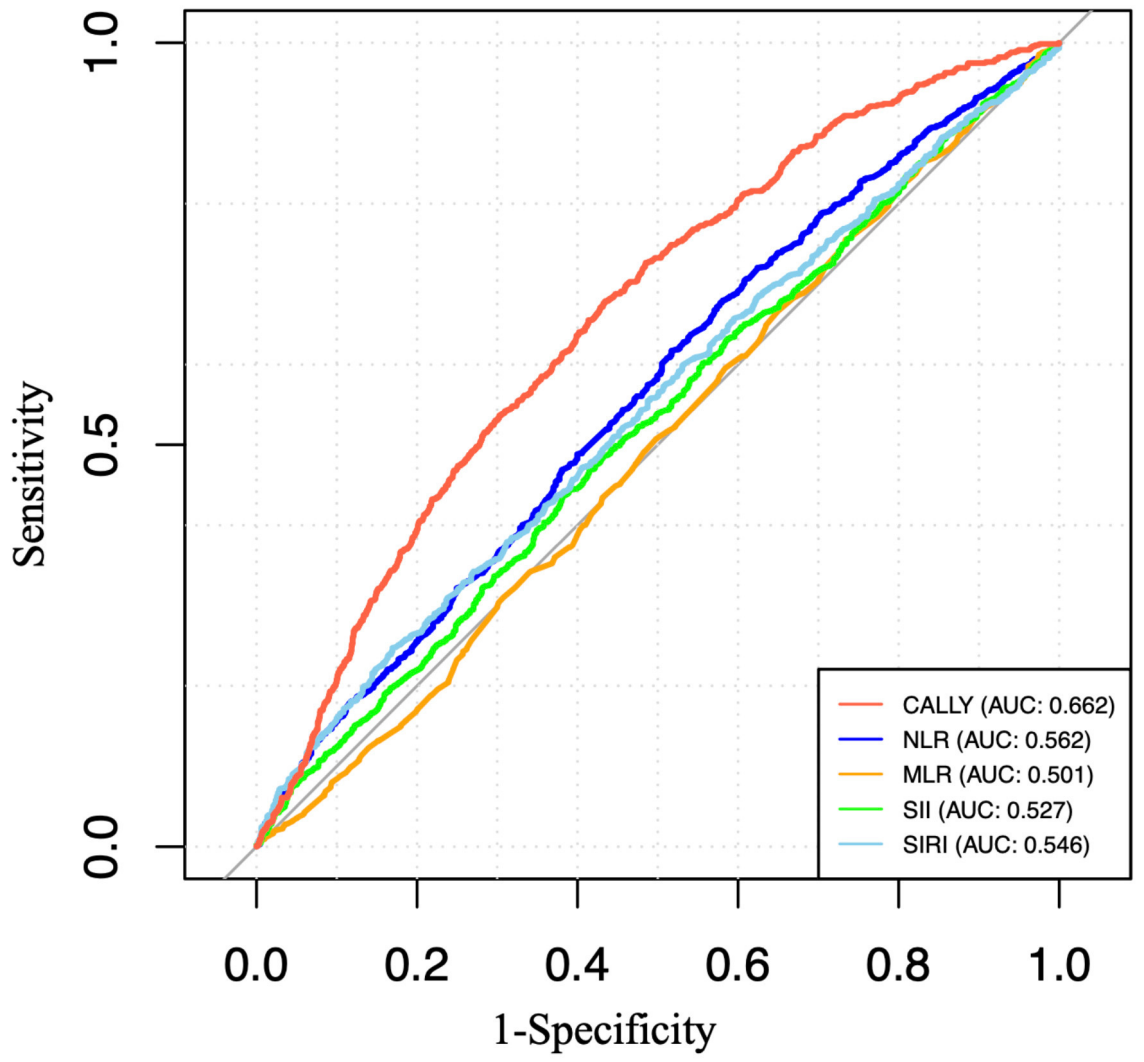
ROC curves and the AUC values of the six inflammatory biomarkers (CALLY, SII, SIRI, NLR, and MLR) in diagnosing retinopathy.

To evaluate and compare the predictive capacity of CALLY with other inflammatory biomarkers, including SII, SIRI, MLR, and NLR, we calculated the area under the curve (AUC) values, as shown in Figure 4. Our findings revealed that CALLY exhibited a higher AUC value compared to the other inflammatory biomarkers.

## 4 Discussion

Using the NHANES database, our study provides novel evidence that CALLY is significantly and inversely associated with the prevalence of retinopathy. Specifically, higher CALLY levels correlate with a reduced risk of retinopathy. This association persists even after adjusting for potential confounding factors, suggesting that CALLY may serve as a predictive marker for the occurrence and progression of retinopathy.

The CALLY index, a novel inflammatory marker, integrates serum albumin levels, lymphocyte counts, and CRP. Albumin, the most abundant protein in serum and a key indicator of nutritional status, possesses potent antioxidant properties that effectively neutralize free radicals (9, 10). Previous studies have suggested that decreased albumin levels may be associated with the development of diabetic retinopathy (11, 12). Li et al., in a cross-sectional study conducted among the Chinese population, identified a potential protective role of serum albumin in patients with diabetes (13). This effect may be attributed not only to its antioxidant properties but also to its critical role in maintaining vascular permeability and stability. By mitigating vascular leakage and edema, serum albumin helps preserve the integrity of the vascular wall, thereby potentially preventing the onset and progression of diabetic retinopathy (2, 14). Immune system dysregulation can lead to damage to retinal vasculature and neural tissues (2, 15). Immune-inflammatory responses are recognized as a pivotal pathogenic mechanism in retinopathy, particularly in DR (16, 17). Chronic hyperglycemia and oxidative stress activate the immune system, triggering a cascade of inflammatory processes. These include the recruitment and activation of immune cells, such as macrophages, T lymphocytes, and neutrophils, within the retinal microenvironment. These cells release pro-inflammatory cytokines (e.g., TNF-α, IL-1β, and IL-6), reactive oxygen species (ROS), and matrix metalloproteinases (MMPs), collectively contributing to endothelial dysfunction, breakdown of the blood-retinal barrier (BRB), and microvascular damage (18–20). CRP a well-established inflammatory biomarker, has been strongly associated with retinopathy (21, 22). CRP can exert direct effects on retinal microvasculature through various mechanisms. Elevated CRP levels induce endothelial cells to secrete additional pro-inflammatory factors, such as IL-6 and TNF-α, and enhance the adhesion of monocytes and neutrophils, exacerbating local inflammation (23). Moreover, CRP is closely linked to, which may lead to BRB disruption and pathological neovascularization (24, 25). Therefore, the CALLY index can be considered a more sensitive indicator of a patient's immune and inflammatory responses.

SII, SIRI, NLR, and MLR are composite inflammatory markers that have garnered significant attention in studies on retinopathy (5, 26). Previous research has primarily focused on these indices, revealing a strong association between elevated composite inflammatory markers and an increased risk of retinopathy (27–29). Retrospective studies have specifically identified a correlation between the MLR and proliferative diabetic retinopathy (PDR). These findings suggest that MLR could serve as a valuable tool for the early prevention and intervention of PDR, highlighting its potential clinical utility in mitigating disease progression (30). Ji et al. reported that the MLR or NLR could mirror the circulating immune status of the host (31). Furthermore, Sena et al. identified the NLR as a rapid and reliable predictive marker for assessing the severity of DR, demonstrating a significant correlation between NLR and DR grading (32). Similarly, Tang et al., in a larger cohort study, reported that higher baseline NLR levels were associated with an increased risk of DR (33). In our study, we compared SII, SIPI, MLR, and NLR among participants. We only found a significant positive correlation between NLR and retinopathy. This discrepancy may arise because previous studies focused exclusively on diabetic retinopathy, whereas our analysis included retinopathy cases not solely attributed to diabetes.

Subgroup analysis revealed that the association between CALLY and retinopathy was stronger in males compared to females. This may be attributed to sex-related differences in immune responses and inflammatory pathways (34). Previous studies have shown that males tend to have higher levels of systemic inflammation (35, 36), which could amplify the role of inflammatory markers like CALLY in retinal diseases. Age is another significant factor, with the association between CALLY and retinopathy being more pronounced in individuals over 60 years old. This is consistent with age-related physiological changes, such as increased oxidative stress, endothelial dysfunction, and impaired vascular repair mechanisms, making the retinal vasculature more vulnerable to inflammation and strengthening the link between CALLY and retinopathy in older adults (37–39). A higher BMI was also associated with a stronger relationship between CALLY and retinopathy. Obesity is often linked to chronic low-grade inflammation and increased oxidative stress, both of which play critical roles in the development of diabetic retinopathy and other retinal diseases (40). The inflammatory environment induced by excess adiposity may enhance the effect of CALLY, suggesting that it may be more sensitive to the inflammatory state in obese individuals. The presence of chronic conditions such as hypertension and diabetes further amplified the association between CALLY and retinopathy. Hypertension has long been recognized as a major risk factor for retinal damage due to its effects on endothelial function and microvascular integrity (41). Similarly, diabetes, particularly in its later stages, promotes a pro-inflammatory state that exacerbates vascular injury and contributes to the development of diabetic retinopathy (42, 43). The significant associations observed in individuals with hypertension and diabetes highlight that CALLY could serve as a valuable marker for monitoring retinal disease progression in these high-risk populations.

Our mediation analysis further revealed that HDL partially mediates the relationship between CALLY and retinopathy. HDL, a key lipid particle, has been recognized for its significant biological role in various cardiovascular and metabolic diseases. HDL primarily functions through reverse cholesterol transport (RCT), removing excess cholesterol from the body and reducing oxidative stress and inflammation, thereby demonstrating its potential to combat atherosclerosis. HDL has also been shown to possess anti-inflammatory properties, with previous studies highlighting its pathogenic role in age-related macular degeneration, potentially related to its ability to regulate complement system activation and inhibition (44). HDL can suppress the activation and migration of monocytes, reducing their accumulation at inflammatory sites. Additionally, HDL promotes the secretion of anti-inflammatory cytokines, such as IL-10, while inhibiting the expression of pro-inflammatory cytokines, including TNF-α and IL-1β, thereby modulating local immune responses (45, 46). Furthermore, HDL exhibits antioxidant properties. Chronic hyperglycemia, lipid peroxidation, and other factors can lead to damage of retinal microvascular endothelial cells, compromising the blood-retinal barrier [BRB; (47, 48)]. As a potent antioxidant, HDL effectively neutralizes free radicals and reactive oxygen species (ROS), thereby mitigating oxidative damage to the retina. HDL also enhances its antioxidant capacity by activating antioxidant enzymes such as superoxide dismutase (SOD) via apolipoprotein A-I (apoA-I) on its surface (49, 50). This antioxidant action of HDL is critical in reducing oxidative damage to retinal blood vessels and neural tissues, positioning it as a promising factor in the prevention and treatment of retinal diseases.

Currently, the CALLY index is primarily used for prognostic prediction in cancer patients (3, 6, 51). It has been independently associated with colorectal cancer outcomes (3). Studies have also shown that the CALLY index is significantly inversely correlated with all-cause and cardiovascular mortality in the older adult (52). In this study, we explored the application of the CALLY index in predicting retinopathy for the first time. An AUC value of 0.672 indicates that the CALLY index has moderate predictive ability in identifying retinopathy. This level of predictive performance still holds clinical relevance, particularly for early screening and risk stratification. Furthermore, when compared to other inflammatory markers such as SII, SIPI, NLR, and MLR, the CALLY index demonstrated superior predictive power, highlighting its potential utility in clinical settings where multiple biomarkers are considered. The CALLY index is a multidimensional nutritional-immune-inflammatory score, incorporating factors such as hypoalbuminemia, which reflects nutritional status and liver function; reduced lymphocyte count, which may indicate immune dysregulation; and elevated CRP levels, signifying increased inflammation. As such, the CALLY index provides a comprehensive reflection of overall health, offering greater stability and being less influenced by various physiological and pathological conditions than individual biomarkers like albumin, monocytes, lymphocytes, and white blood cell count. This makes it a promising tool for assessing disease risk across multiple domains.

This study has several limitations. First, due to its retrospective design, potential biases cannot be entirely ruled out, and the cross-sectional nature of the study restricts the ability to infer causality between the CALLY index and retinal diseases. Second, while we adjusted for multiple variables, the possibility of unaccounted confounding factors (such as medication use) remains. Lastly, although our study focused on all types of retinopathy, it did not further investigate the differential performance of the CALLY index across various retinopathy subtypes. Future research should track longitudinal changes in the CALLY index and explore its relationship with the development of retinopathy. Additionally, stratified analyses based on diabetic retinopathy and hypertensive retinopathy should be conducted to further elucidate the association between the CALLY index and retinopathy.

Our findings offer several important implications for future research. First, prospective cohort studies are needed to establish the causal and dynamic relationship between the albumin-lymphocyte-CRP factor and the progression of retinopathy. Second, further investigation is required to explore the association between the CALLY index and retinopathy caused by different underlying etiologies, as well as to evaluate the sensitivity and specificity of the CALLY index across different ethnic groups and populations. Third, clinical data-based studies are necessary to comprehensively assess the predictive and practical value of the CALLY index. In parallel, in-depth research into the pathophysiological mechanisms is crucial for identifying potential therapeutic targets and biomarkers.

From the perspective of chronic disease management, these findings highlight the value of integrating the CALLY index into digital medical records for the early prediction of retinopathy in patients with hypertension and diabetes. This approach underscores the potential to advance the comprehensive integration of prevention, treatment, and management of chronic diseases and their complications, reducing the economic burden on patients with chronic conditions.

## 5 Conclusions

This study highlights a significant inverse correlation between the CALLY index and retinopathy, demonstrating its superiority over traditional inflammatory markers as a reliable and independent predictor of retinopathy. These findings underscore the potential clinical utility of the CALLY index as a tool for early identification of individuals at risk for retinopathy, particularly in the long-term management of patients with hypertension and diabetes.

## Data Availability

The raw data supporting the conclusions of this article will be made available by the authors, without undue reservation.
