# Supplementary figures and images for: The association between the C-reactive protein-to-albumin-to-lymphocyte index and retinopathy: insights from a population-based study
Source: Front Nutr. 2025 Mar 13;12:1552020. doi: 10.3389/fnut.2025.1552020 (PMC11948663; doi:10.3389/fnut.2025.1552020)

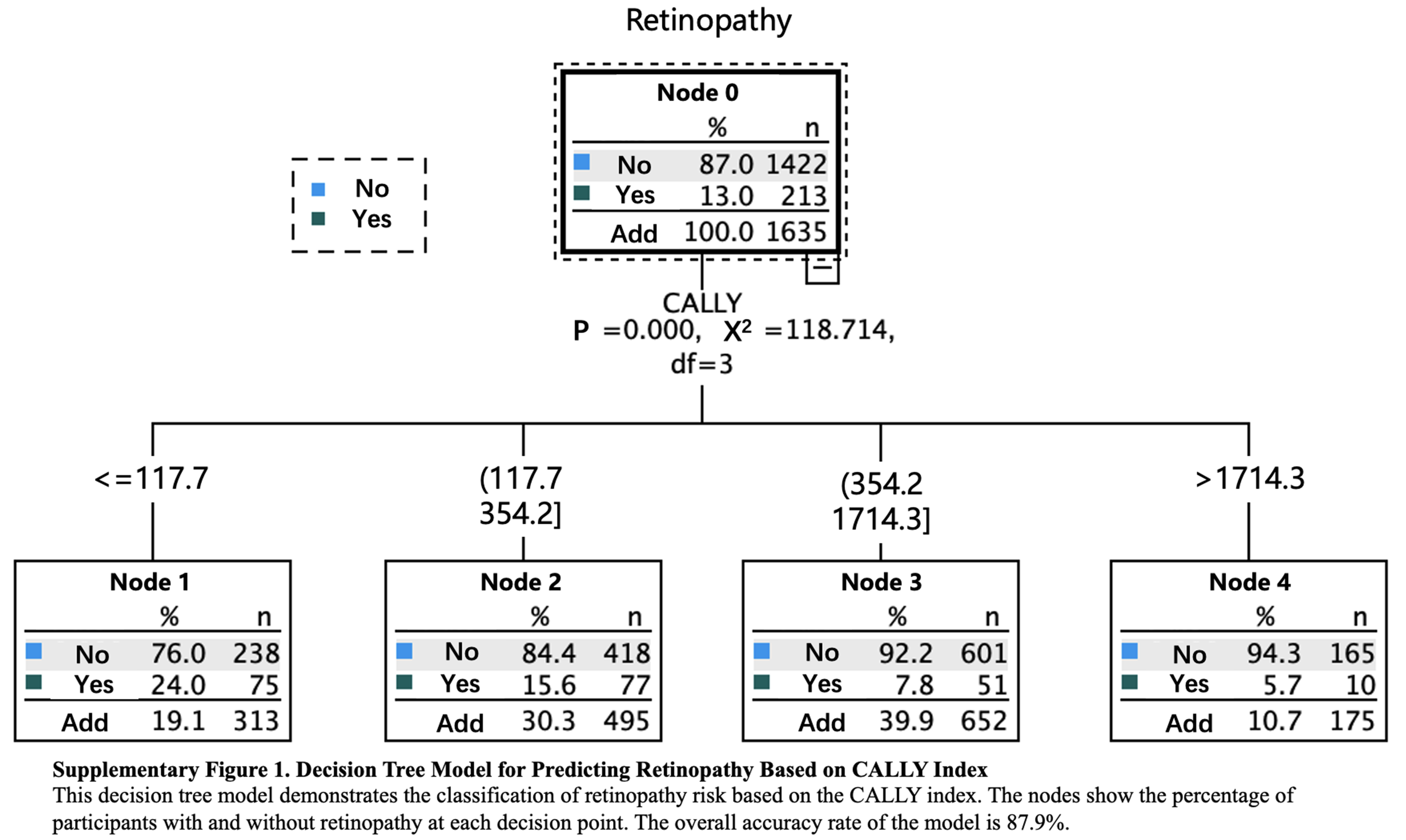

Supplement: Supplementary file 2 [file Image_1.png]

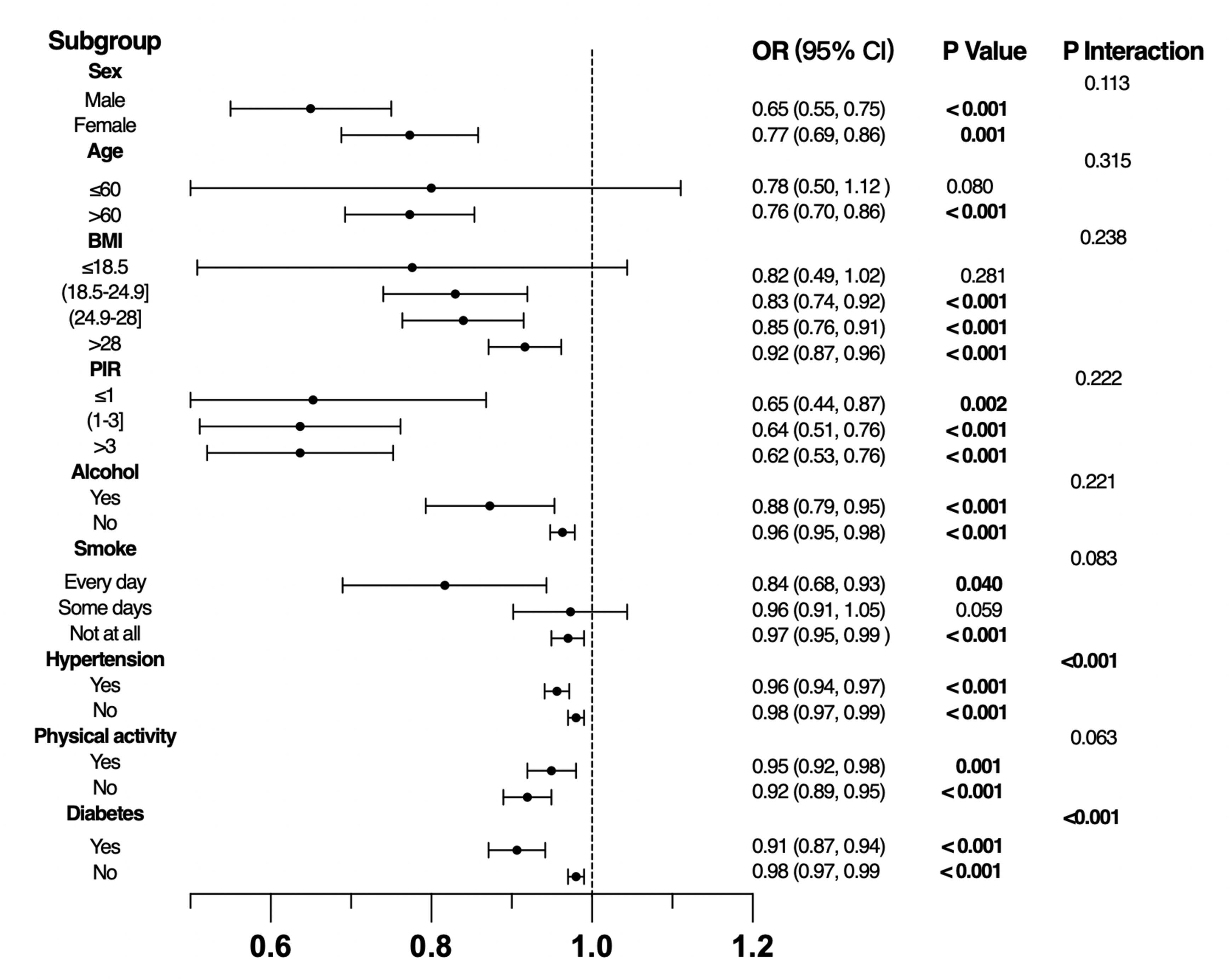

Supplement: Supplementary file 3 [file Image_2.png]
